# Новые генетические технологии защиты растений от паразитических нематод

**DOI:** 10.18699/VJ21.037

**Published:** 2021-05

**Authors:** A.V. Kochetov, T.A. Gavrilenko, O.S. Afanasenko

**Affiliations:** Institute of Cytology and Genetics of the Siberian Branch of the Russian Academy of Sciences, Novosibirsk, Russia; Institute of Cytology and Genetics of the Siberian Branch of the Russian Academy of Sciences, Novosibirsk, Russia Federal Research Center the N.I. Vavilov All-Russian Institute of Plant Genetic Resources (VIR), St. Petersburg, Russia; Institute of Cytology and Genetics of the Siberian Branch of the Russian Academy of Sciences, Novosibirsk, Russia All-Russian Institute of Plant Protection, Pushkin, St. Petersburg, Russia

**Keywords:** cyst nematodes, gall nematodes, potato, effector genes, R-genes, RNA interference, host induced genetic silencing, цистообразующие нематоды, галловые нематоды, картофель, гены-эффекторы, R-гены, РНКинтерференция, хозяин-индуцированный генетический сайленсинг

## Abstract

Нематоды относятся к числу значимых вредителей сельскохозяйственных растений. В обзоре рассмотрены последние данные о молекулярных механизмах устойчивости растений к цистообразующим и галловым нематодам, среди которых одни из наиболее вредоносных видов: Globodera rostochiensis, G. pallida, Heterodera schachtii, Meloidogyne chitwoodi и M. incognita. Например, золотистая картофельная нематода G. rostochiensis, зарегистрированная в 61 субъекте РФ на общей площади 1.8 млн га, способна приводить к потере от 19 до 90 % урожая картофеля. Биологические особенности нематод затрудняют разработку агротехнических способов борьбы с ними: цисты G. rostochiensis сохраняют жизнеспособность в почве в течение многих лет, нематициды токсичны или малоэффективны, поэтому предпочтительным методом борьбы с ними является интрогрессия генов устойчивости от родственных культурных и дикорастущих видов. Стратегия жизненного цикла цистообразующих и галловых нематод основана на способности личинок проникать в корни восприимчивых видов растений, репрограммировать клетки растения-хозяина, формирующие гигантские клетки или синцитии в качестве питающих структур, а также ингибировать иммунный ответ. Молекулярные механизмы, лежащие в основе такого взаимодействия в системе «патоген–хозяин», вызывают значительный интерес как с точки зрения управления морфогенезом растений, так и в аспекте разработки безопасных и эффективных способов борьбы с паразитическими нематодами. В обзоре рассмотрены данные об эффекторах, с помощью которых разные виды нематод контролируют иммунный ответ растения-хозяина, а также гены устойчивости (R-гены) и некоторые молекулярные механизмы, прерывающие формирование питающих структур и развитие паразита. Приведены новые данные о способах генетического контроля, основанных на одном из активно обсуждаемых в последнее время варианте механизма РНК-интерференции – HIGS (host induced gene silencing), представляющем собой адресное выключение экспрессии гена-мишени в клетках личинки нематоды с помощью специфических двуцепочечных РНК, синтезирующихся в клетках растения-хозяина. Индукция РНК-интерференции в клетках растений приводит к появлению молекул-медиаторов, способных инициировать аналогичный процесс в клетках фитофагов, взаимодействующих с растением, в том числе у личинок нематод. Описаны случаи, в которых такое адресное выключение экспрессии генов-мишеней приводило к нарушениям развития личинок и высокому уровню защиты сельскохозяйственных растений от наиболее опасных видов нематод.

## Abstract (en)

. Nematodes belong to economically important pests. Here we reviewed the recent data on molecular mechanisms of plant resistance to cyst and gall nematodes including the most devastating Globodera rostochiensis, G. pallida, Heterodera schachtii, Meloidogyne chitwoodi, and M. incognita. The Golden Potato Cyst Nematode (G. rostochiensis, GPCN) may be taken as an example of an economically important pest: in Russia, it occurs in 61 regions with a total area of 1.8 million ha and may cause the yield loss from 19 to 90 %. The biological characteristics of sedentary nematodes makes their agrotechnical control problematic, i.e. the GPCN cysts remain dormant in soil for many years until a susceptible host appears, whereas nematicides are either toxic or inefficient. Introgression of resistance genes (R-genes) from related cultivated or wild species is likely to be the most appropriate way for their biocontrol. The life cycle of sedentary nematodes is based on juveniles’ penetration into the host root where they reprogram plant cells into a syncytium or the so-called ‘giant cells’ and inhibit the plant defense response. Molecular mechanisms of plant-nematode interaction are unusual and this phenomenon provides a very interesting model for the investigation of plant morphogenesis control as well as for the development of new genetic instruments of biocontrol. Here we reviewed recent publications on plant parasitic nematode effectors used for hijacking of the plant immune system, data on R-genes and molecular mechanisms of their activities. In addition, host-induced gene silencing (HIGS) is discussed as a perspective mechanism for nematode biocontrol. HIGS is based on the RNA interference in the cells of the host plant addressed against the nematode genes important for their development and productivity. Several recent investigations demonstrated efficiency of HIGS against sedentary nematodes.

## Введение

Фитопаразитические нематоды относятся к числу значимых вредителей сельского хозяйства и распространены по всему миру (около 4 тыс. видов, большинство из которых встречаются в странах с тропическим и субтропическим климатом) (Зиновьева и др., 2012). Из многочисленных видов фитопаразитических нематод в обзоре наибольшее внимание уделено цистообразующим и галловым, поскольку к ним относятся виды, наносящие значительный урон экономически важным сельскохозяйственным культурам растений, среди которых Globodera rostochiensis (Woll), G. pallida (Stone), Heterodera schachtii (Schmidt), Meloidogyne chitwoodi (Golden et al.), Meloidogyne incognita (Kofoid & White). Следует отметить, что M. incognita называют наиболее опасным и вредным организмом, поражающим культурные растения (Trudgill, Blok, 2001)

Разработка способов борьбы с паразитическими нематодами считается одной из актуальных биотехнологических задач, однако их биологические особенности затрудняют применение типовых агротехнических и химических методов защиты. Примером может служить золотистая картофельная нематода (ЗКН) G. rostochiensis – широко распространенный карантинный вредитель, приводящий к существенным потерям в картофелеводстве (Evans, Trudgill, 1992). В России ЗКН зарегистрирована в 61 субъекте, включающем 861 административный район на территории общей площадью 1.8 млн га (Справочник по карантинному фитосанитарному состоянию, 2017). В зависимости от сорта и сезонных условий потери урожая картофеля могут составлять от 19 до 90 % (Friedman, 1985), при этом покоящиеся в почве цисты ЗКН способны сохранять жизнеспособность более 30 лет (Winslow, Willis, 1972). Кроме того, следует учитывать высокую вероятность распространения ЗКН в новых, не характерных для этого вида регионах вследствие прогнозов по изменению климата (Jones et al., 2017), а также возможности появления на территории РФ других опасных для картофелеводства карантинных видов нематод

Способы борьбы с ЗКН включают использование нематицидов (Kearn et al., 2017) и применение в севообороте родственных культур, способных инициировать выход личинок ЗКН, но не являющихся для них хозяевами, что приводит к гибели личинок и снижению инфицированности почвы (например, Solanum sisymbriifolium) (Dandurand et al., 2019; Kooliyottil et al., 2019). Однако эти методы могут быть недостаточно результативны или экологичны: так, эффективные нематициды токсичны и запрещены в европейских странах (Trudgill et al., 2003). Таким образом, предпочтительным подходом к контролю ЗКН в разных странах является возделывание устойчивых сортов с интрогрессированными генами устойчивости (R-генами) от родственных культурных (S. tuberosum ssp. andigenum) и дикорастущих (S. canasense, S. oplocense, S. spegazzinii, S. vernei) видов картофеля (Dalamu et al., 2012).

Молекулярные механизмы патогенеза также превращают ЗКН в непростую мишень для существующих способов защиты растений. Личинки ЗКН после выхода из яиц в почве проникают в корни восприимчивых растений, движутся до проводящих тканей и инициируют формирование питающего синцития. Для этого личинка ЗКН впрыскивает в клетки растения секрет из пищеводных желез, инициирующий репрограммирование и слияние клеток в синцитий (описаны синцитии, образовавшиеся в результате последовательного слияния более чем 200 клеток), а также ингибирующий иммунный ответ у восприимчивых сортов (Mejias et al., 2019). Сходный механизм формирования системы питания выработали галлообразующие нематоды семейства Meloidogynidae (мелойдогинид), также способные репрограммировать клетки растенияхозяина, инициировать процесс митоза без цитокинеза с формированием так называемых гигантских клеток, размер которых может более чем в 300 раз превышать исходную клетку. Личинка нематоды в данном случае формирует специализированный орган – галл, в котором может быть несколько гигантских клеток (Зиновьева и др., 2012; Mejias et al., 2019). Оба типа питающих структур (синцитий и гигантские клетки) характеризуются общими структурно-функциональными особенностями: развитый эндоплазматический ретикулум, утолщенные клеточные стенки, большое количество митохондрий, фрагментированная вакуоль и т.п. (Rodiuc et al., 2014; Palomares-Rius et al., 2017; Mejias et al., 2019). Механизмы индуцированного нематодами репрограммирования активно исследуют, так как этот биологический феномен представляет собой перспективную модель для изучения генетического контроля морфогенеза растений.

Известно, что личинки южной галловой нематоды Meloidogyne incognita секретируют сложный набор веществ различной природы, содержащий не менее 500 белков, функции большинства из которых неизвестны (Wang et al., 2012). В настоящее время можно выделить несколько PPN-эффекторов (plant parasitic nematode effectors), о функциях которых есть экспериментальные данные.

**PPN-эффекторы, способные связывать активные формы кислорода (АФК), которые образуются в результате сверхчувствительной реакции.** Эффективные способы борьбы с патогенами, выработанные растениями в ходе эволюции, включают распознавание специфических молекулярных паттернов (pathogen-associated molecular patterns, PAMP), индукцию системного защитного ответа и локально – программируемой клеточной смерти в месте инвазии. В случае личинок нематод зона мертвых клеток блокирует поступление питательных веществ и останавливает жизненный цикл. Известно, что АФК являются одним из ключевых медиаторов в регуляторных контурах, контролирующих этот вид иммунного ответа (effector-triggered immunity, ETI). Показано, что один из PPN-эффекторов яванской галловой нематоды Meloidogyne javanica (Treub, 1885) взаимодействует с каталитической субъединицей тиоредоксинредуктазы и связывает АФК, что блокирует передачу сигнала и ингибирует развитие иммунного ответа (Lin et al., 2016). Другую клеточную систему используют для этой же цели личинки свекловичной цистообразующей нематоды Heterodera schachtii: эффектор Hs10A06 связывается со спермидинсинтазой, что увеличивает синтез спермидина, также связывающего АФК (Hewezi et al., 2015). 

**PPN-эффекторы, ингибирующие локальный и системный иммунный ответ.** Помимо связывания АФК PPN-эффекторы способны инактивировать PR-белки и ингибировать системный иммунный ответ, в частности в качестве мишеней могут быть использованы ферменты бета-1,3-эндоглюканазы, 1,3-бета-глюкансинтазы и др. (Mejias et al., 2019). Для ингибирования специфического иммунитета можно применять инактивацию специфического белка-рецептора: например, PPN-эффектор G. pallida RHA1B является убиктвитинлигазой, способной как активировать убиквитинилирование и протеолиз белкарецептора семейства NBS-LRR, так и ингибировать индукцию специфического иммунитета в целом, например в ответ на молекулярные паттерны других патогенов (Kud et al., 2019). 

**PPN-эффекторы, способные ингибировать иммунный ответ на уровне молекулярно-генетических регуляторных контуров.** В качестве примера можно привести эффектор M. incognita, взаимодействующий с одной из субъединиц сигналосомы COP9 (Bournaud et al., 2018). Характерно, что этот белок, участвующий в пути передачи сигнала салициловой кислоты, также является мишенью для ряда эффекторов грибов и вирусов. Аналогичным образом PPN-эффекторы сразу нескольких видов нематод (M. chitwoodi, G. rostochiensis, H. schachtii) используют в качестве мишени белок PLCP (papain-like cysteine protein), который также служт одной из общих мишеней для различных патогенов (Mejias et al., 2019)

**PPN-эффекторы, задействованные в индукции формирования синцития или гигантских клеток.** Про функции этих белков известно немного. Цистообразующая бледная картофельная нематода G. pallida продуцирует эффектор GpSPRY-414-2, который связывается с белком CLASP, ассоциированным с микротрубочками и участвующим в делении и росте клеток (Mei et al., 2018); вероятно, эту мишень используют для реорганизации цитоскелета в синцитии. Можно отметить PPN-эффекторы цистообразующих нематод, структура которых сходна с CLAVATA3 (CLV3)/ESR (CLE) – регуляторными пептидами, задействованными в контроле деления и дифференцировки клеток растений, а также эффекторы H. schachtii, взаимодействующие с транспортером ауксина LAX3 (Gheysen, Mitchum, 2019). Тема репрограммирования клеток растения-хозяина тесно связана и с вопросом специфичности нематод: помимо преодоления иммунной системы важным элементом жизненного цикла патогена является адресное воздействие на системы молекулярно-генетического управления морфогенезом, что также относится к актуальным направлениям исследований (Sabeh et al., 2019).

## Гены устойчивости (R-гены)

Гены устойчивости к G. rostochiensis свидетельствуют о том, что в настоящее время большинство сортов картофеля содержат по одному R-гену (Whitworth et al., 2018), обеспечивающему высокую устойчивость к патотипу ЗКН Ro1. К их числу относятся H1, интрогрессированный от S. tuberosum ssp. andigenum (Bakker et al., 2004), и Gro1-4 от S. spegazzinii (Barone et al., 1990; Ballvora et al., 1995). Защита, контролируемая идентифицированными генами устойчивости, основана на механизме реакции сверхчувствительности: индукция программируемой клеточной смерти формирует зону мертвых клеток, отсекающих личинку ЗКН от поступления питательных веществ, что не позволяет ей завершить жизненный цикл (Kaloshian et al., 2011; Зиновьева и др., 2012; Kochetov et al., 2017). Несмотря на то что гены H1 и Gro1-4 сохраняют эффективность в Европе и России, следует учитывать, что нематоды способны к быстрой эволюции, поэтому поиск новых генов устойчивости к ЗКН считается одной из приоритетных задач генетики растений (Whitworth et al., 2018; Strachan et al., 2019). Одним из классических способов расширения набора генов устойчивости с перспективами их дальнейшего пирамидирования является поиск новых генов в природных популяциях растений. В качестве примера можно привести систематический скрининг биоресурсных коллекций ВИР, показавший наличие нового R-гена для G. rostochiensis у некоторых генотипов S. phureja (Limantseva et al., 2014). Сравнительный анализ транскриптомов устойчивых и восприимчивых генотипов позволил определить механизм устойчивости, основанный на индукции реакции сверхчувствительности и локальной программируемой клеточной смерти в зоне инвазии личинок. Особенностями взаимодействия нематод с корнями растений служат существенное повреждение тканей при миграции личинок и индукция неспецифического ответа на этот вид стресса (wounding), который у устойчивых линий дополняется специфическим PAMP-опосредованным иммунным ответом в виде реакции сверхчувствительности и системного синтеза защитных белков (Kochetov et al., 2017, 2020)

Подобный подход использован для изучения генетических механизмов устойчивости к галловым нематодам различных экономически важных видов пасленовых, например картофеля (Bali et al., 2019), томата (Schaff et al., 2007) и табака (Li et al., 2018). В работе S. Bali и коллег исследован селекционный клон с интрогрессированным из дикого диплоидного мексиканского вида картофеля S. bulbocastanum геном RMC1(bulb), обеспечивающим устойчивость к Meloidogyne chitwoodi. Аналогично данным, представленным A.V. Kochetov и сотрудниками (2017, 2020), личинки нематоды проникают в корни как восприимчивых, так и устойчивых сортов растений, однако PAMP-опосредованная реакция сверхчувствительности ограничивает формирование питающих гигантских клеток, и жизненный цикл нематоды прерывается. Развитие иммунного ответа приводит к масштабным изменениям в транскриптоме, что позволяет выявлять дифференциально экспрессирующиеся гены у восприимчивых и устойчивых генотипов, реконструировать системы взаимодействующих генов (генные сети), пути передачи сигнала и молекулярные механизмы устойчивости. Показано, что индукция устойчивости связана с накоплением АФК, увеличением активности генов сигнальных путей жасмоновой и салициловой кислот, синтеза компонентов клеточной стенки, полиаминов, PR-белков (Bali et al., 2019). X. Li и коллеги исследовали взаимодействие устойчивых и восприимчивых генотипов Nicotiana tabacum с M. incognita. На этой модели также продемонстрировано, что личинки нематод проникают в корни как устойчивых, так и восприимчивых растений, но у первых на седьмой день инициируются реакция сверхчувствительности и локальный некроз в районе гигантских питающих клеток. В результате сравнительного анализа транскриптома и дифференциально экспрессирующихся генов выявлены путь передачи сигнала салициловой кислоты, гены антиоксидантов, а также метаболические пути синтеза фенилпропаноидов, алкалоидов и терпеноидов, что может быть характерно для табака (Li et al., 2018).

Несмотря на то что защитные механизмы часто связаны со специфическими рецепторами и индукцией реакции сверхчувствительности, в основе иммунного ответа могут быть разные молекулярные события. Можно отметить, что разнообразие защитных механизмов в природных популяциях растений изучено недостаточно и новые варианты генов устойчивости могут отличаться от классических рецепторов семейства NBS-LRR, активирующих реакцию сверхчувствительности при появлении нематодспецифических молекулярных паттернов. Например, ген Rhg1 сои, кодирующий специфический вариант белка везикулярного транспорта α-SNAP (Bayless et al., 2018), способен интерферировать с молекулярными механизмами патогенеза цистообразующих нематод, однако при этом его экспрессия в трансгенных растениях других видов (картофеля, арабидопсиса, других разновидностей сои) приводила как к увеличенной устойчивости к H. schachtii, G. rostochiensis и G. pallida, так и негативно влияла на рост и развитие растений (Butler et al., 2019). По-видимому, в процессе эволюции у устойчивых форм сои сформировался баланс в экспрессии генов, кодирующих белки α-SNAP, обеспечивающий устойчивость к патогену и компенсацию негативного эффекта такого необычного R-гена. 

## Новые способы генетического контроля устойчивости растений к нематодам

В последнее время значительный интерес вызывает один из вариантов технологии РНК-интерференции, а именно HIGS (host induced gene silencing), представляющий собой адресное выключение экспрессии гена-мишени в клетках личинки нематоды с помощью специфических двуцепочечных РНК (дцРНК), синтезирующихся в клетках растения-хозяина. дцРНК способны индуцировать систему защитного ответа на основе РНК-интерференции, приводящую к появлению коротких интерферирующих РНК (short interfering RNA, siRNA) и комплексов RISC, способных распознавать и разрушать мишени – молекулы РНК, содержащие участки, идентичные или высокогомологичные такой дцРНК-затравке. Этот механизм является не только способом борьбы с вирусами, но и одним из фундаментальных молекулярных механизмов контроля экспрессии генов у эукариот. Однако дцРНК, siRNA или другие промежуточные формы процесса РНКинтерференции могут проникать в клетки организмов, взаимодействующих с растением, например в клетки пищеварительной системы насекомых-фитофагов, гифы паразитических грибов, клетки нематод и др. При этом если дцРНК сконструирована из сегментов мРНК, соответствующих не гену растения-хозяина, а гену фитофага, то в ряде случаев при взаимодействии этого фитофага с растением отмечены индукция РНК-интерференции и специфическое ингибирование экспрессии гена-мишени в клетках фитофага. Если данный ген выполнял жизненно важные функции, то растения, продуцирующие такие дцРНК, становились токсичными для соответствующего вредителя.

Указанный биологический феномен демонстрирует возможность обмена регуляторной генетической информацией между организмами различной таксономической принадлежности в природных и искусственных экосистемах, что требует дальнейшего всестороннего исследования. Тем не менее очевидно и прикладное значение феномена HIGS, так как дцРНК, специфические для мРНК конкретного гена-мишени, не оказывают воздействия на организмы, в транскриптомах которых нет мРНК с протяженными участками сходства. В структуре мРНК эукариот помимо белок-кодирующей части (открытой рамки считывания, CDS) выделяют 5′- и 3′-концевые нетранслируемые последовательности (НТП). 5′-НТП играет важную роль в контроле инициации трансляции, в то время как функции 3′-НТП могут быть связаны с управлением цитоплазматической стабильности индивидуальных мРНК (Кочетов и др., 2002; Kochetov, Sarai, 2004; Ventoso et al., 2012). В качестве адресной нуклеотидной последовательности для индукции РНК-интерференции при конструировании дцРНК можно использовать более протяженные 3′-НТП мРНК гена-мишени, которые, в отличие от белок-кодирующих участков, в большинстве случаев не являются эволюционно консервативными, что расширяет диапазон возможностей селективного видоспецифического выключения отдельных генов. 

Следует отметить, что эффективность воздействия дцРНК может зависеть от морфологических и биохимических особенностей организма-мишени, в частности включающих барьеры на пути проникновения дцРНК внутрь клеток и содержание в тканях дцРНК-специфических рибонуклеаз. При этом не все гены организма-реципиента могут быть супрессированы с помощью РНК-интерференции. Так, в работе S. Iqbal и коллег (2020) приведены результаты систематического исследования генов домашнего хозяйства M. incognita, которые в перспективе могут быть использованы как мишени для HIGS. Выбор подходящего гена-мишени – одна из важных задач, так как некоторые гены домашнего хозяйства могут быть малочувствительны к РНК-интерференции либо эффект от их супрессии может быть недостаточно выражен. Авторы использовали методику, адаптированную из ранних экспериментов на Caenorhabditis elegans, – вымачивание личинок нематоды в растворе, содержащем дцРНК, и последующий анализ характеристик, включая инфекционную способность личинок и характеристики взрослых нематод после инфицирования личинками растений томата. Проанализировано 20 генов: экспрессия восьми генов не репрессировалась дцРНК и не вызывала морфологических проявлений, в то время как супрессия десяти генов приводила к аберрациям в морфологических характеристиках нематод и снижению их способности к инфицированию растений. Трансгенные растения, экспрессирующие дцРНК против шести генов нематоды, в экспериментах проявляли устойчивость, сопоставимую с присутствием у растений R-генов: снижение инфицированности растений доходило до 89 % (Iqbal et al., 2020).

Далее приведены данные успешного применения технологии HIGS как потенциального инструмента борьбы с паразитическими нематодами. Интересные результаты получены при попытке использования HIGS в качестве мишени гена M. incognita, кодирующего PPN-эффектор Mi-MSP2, участвующий в супрессии защитного ответа растения-хозяина. Трансгенные растения, экспрессирующие такие дцРНК, характеризовались сниженной на 88 % экспрессией гена-мишени у самок нематоды, а также уменьшенной на 80 % продукцией яиц (Joshi et al., 2019). Применение этой технологии для защиты растений баклажана показало, что дцРНК стабильно экспрессировалась в трансгенных формах вплоть до третьего поколения от самоопыления и обеспечивала высокий уровень защиты от нематоды (Chaudhary et al., 2019). Для M. incognita, одного из наиболее опасных вредителей растений, обладающего широким кругом хозяев, экспериментально доказана эффективность HIGS для нескольких генов, включая PPN-эффекторы (Shivakumara et al., 2017; снижение продуктивности самок нематоды составило 40–70 %), ген стерол-связывающего белка (Shivakumara et al., 2019; снижение продуктивности нематод до 50 %), ген L-цистеиновой протеазы (Dutta et al., 2015; снижение продуктивности на 60–80 %), гены кутикулярного коллагена (Banerjee et al., 2018; снижение продуктивности нематод до 80 %, структурные аберрации у личинок).

Развитие других видов нематод также может быть супрессировано с помощью HIGS. Отмечено снижение продуктивности самок H. schachtii на трансгенных растениях, экспрессирующих дцРНК сложной структуры, содержащую сегменты генов эндоглюканазы и белка MSP (major sperm protein) (Amin et al., 2018); кроме того, показана возможность применения HIGS для Heterodera glycines (Hu et al., 2019), Bursaphelenchus xylophilus (Qiu et al., 2019) и других видов нематод. Свекловичная цистообразующая нематода H. schachtii также служит примером актуальности разработки новых генетических технологий биологического контроля. Этот паразит поражает более 200 видов растений, принадлежащих 98 родам, и является одним из основных вредителей сахарной свеклы. Яйца в цистах сохраняют жизнеспособность в почве в течение нескольких лет. Стандартные способы борьбы с помощью нематицидов на основе фосфоорганических соединений и карбаматов затруднены из-за их высокой токсичности; применение в севообороте других видов растений, способных инициировать выход личинок из яиц, но не являющихся хозяевами для H. schachtii, экономически неэффективно для высокоинтенсивных технологий возделывания сахарной свеклы. Использование устойчивых форм растений также проблематично из-за их повышенной чувствительности к некоторым грибным патогенам (Amin et al., 2018), что в совокупности демонстрирует отсутствие эффективных способов борьбы и необходимость разработки новых технологий, к числу которых относится HIGS.

## Заключение

Применение технологии HIGS основано на получении генетически-модифицированных форм растений, которые не экспрессируют чужеродные белки, но производят некодирующую двуцепочечную РНК, содержащую участки мРНК гена-мишени, в данном случае гена нематоды. РНК-интерференцию, как и белок-кодирующие гены, активно применяют для супрессии генов растений (Кочетов и др., 2004; Trifonova et al., 2007; Sugawara et al., 2016). Поскольку при продукции некодирующей дцРНК не производятся новые для растения-хозяина белки, отсутствует вероятность развития у потребителей аллергических реакций или специфических изменений в метаболоме растений, связанных с новыми ферментативными или регуляторными активностями. К рискам применения таких растений в практике сельского хозяйства следует отнести потенциальную возможность неспецифического действия дцРНК на другие организмы, взаимодействующие с растениями, если их мРНК каких-либо генов содержат протяженные участки сходства. Дальнейшее систематическое исследование структуры геномов организмов разной принадлежности в различных природных и агроэкосистемах, вероятно, позволит с точностью прогнозировать такие риски и откроет путь к инженерии устойчивости сельскохозяйственных растений к разнообразным вредителям и патогенам

## Conflict of interest

The authors declare no conflict of interest.
